# Reduced CCR5 expression among Uganda HIV controllers

**DOI:** 10.1186/s12977-023-00626-7

**Published:** 2023-05-25

**Authors:** Brian Nyiro, Sharon Bright Amanya, Alice Bayiyana, Francis Wasswa, Eva Nabulime, Alex Kayongo, Immaculate Nankya, Gerald Mboowa, David Patrick Kateete, Obondo James Sande

**Affiliations:** 1 New Jersey Medical School, Rutgers University, New Jersey, USA; 2 Baylor College of Medicine, Houston, TX USA; 3 Department of Immunology and Molecular Biology, Makerere University, Kampala, Uganda; 4 Makerere University Lung Institute, Kampala, Uganda; 5 Centre for AIDS Research Laboratory, Joint Clinical Research Centre, Wakiso, Uganda

**Keywords:** Elite controllers, Viremic controllers, Non-controllers, HIV, CCR5 promoter polymorphisms

## Abstract

**Background:**

Several mechanisms including reduced CCR5 expression, protective HLA, viral restriction factors, broadly neutralizing antibodies, and more efficient T-cell responses, have been reported to account for HIV control among HIV controllers. However, no one mechanism universally accounts for HIV control among all controllers. In this study we determined whether reduced CCR5 expression accounts for HIV control among Ugandan HIV controllers. We determined CCR5 expression among Ugandan HIV controllers compared with treated HIV non-controllers through *ex-vivo* characterization of CD4 + T cells isolated from archived PBMCs collected from the two distinct groups.

**Results:**

The percentage of CCR5 + CD4 + T cells was similar between HIV controllers and treated HIV non-controllers (ECs vs. NCs, P = 0.6010; VCs vs. NCs, P = 0.0702) but T cells from controllers had significantly reduced CCR5 expression on their cell surface (ECs vs. NCs, P = 0.0210; VCs vs. NCs, P = 0.0312). Furthermore, we identified rs1799987 SNP among a subset of HIV controllers, a mutation previously reported to reduce CCR5 expression. In stark contrast, we identified the rs41469351 SNP to be common among HIV non-controllers. This SNP has previously been shown to be associated with increased perinatal HIV transmission, vaginal shedding of HIV-infected cells and increased risk of death.

**Conclusion:**

CCR5 has a non-redundant role in HIV control among Ugandan HIV controllers. HIV controllers maintain high CD4 + T cells despite being ART naïve partly because their CD4 + T cells have significantly reduced CCR5 densities.

**Supplementary Information:**

The online version contains supplementary material available at 10.1186/s12977-023-00626-7.

## Introduction

CCR5 is not only a chemokine receptor expressed by several immune cells, including T cells, macrophages, and dendritic cells, but it is also required by the R5 HIV tropic virus to infect CD4 + T cells [1–3]. Mutations in the gene encoding CCR5 for example, the $\varDelta$ 32 bp deletion, have been associated with either delayed progression to AIDS or resistance to HIV infection [4–9]. This knowledge has led to the development of drugs that competitively bind CCR5 to reduce viral entry and CD4 + T cell depletion [10–12]. Understanding host-pathogen interaction and deciphering immune correlates of protection is vital to identifying therapeutic and drug design targets. This study determined whether CCR5 accounts for HIV control among Ugandan HIV controllers. These individuals have the intrinsic capacity to delay HIV progression to AIDS without antiretroviral therapies. Generally, mechanisms underlying HIV control among controllers remain unknown and those reported vary among different populations.

HIV controllers have been reported in Uganda but the mechanisms for their HIV control remain largely unknown [13–15]. Variations in the expression of several host factors including HLA, CCR5, and viral restriction factors, have been reported as underlying factors associated with this phenotype [16–20]. However, mechanisms for HIV control are heterogeneous with no one mechanism accounting for HIV control among all controllers. HLA, for example, only accounts for approximately 20% of HIV controllers with some individuals with the perceived protective HLA progressing rapidly to AIDS. Furthermore, in 2020, Amanya and colleagues reported that a subset of Ugandan HIV controllers carry the rs10838525 SNP (R136Q variant) within the *Trim 5a* encoding gene [21]. TRIM 5a is a viral restriction factor that interferes with HIV decapsidation [22]. This mutation enhances the protein’s affinity for HIV-1 and recent reports show it may be superior at blocking HIV-1 infection compared to the wild type [23]. However, it only accounted for a subset of HIV controllers.

Our study determined whether reduced CCR5 accounts for HIV control among Ugandan HIV controllers. Previous studies have reported that reduced CCR5 expression protects CD4 + T cells from infection by the R5 tropic virus impairing viral entry and replication thus impeding HIV progression [6, 24]. Despite being ART-naive, HIV controllers maintain comparable CCR5 + CD4 + T cells to treated HIV non-controllers. However, through ex-vivo characterization of CD4 + T cells from HIV controllers, we show that controllers have significantly reduced CCR5 expression which could partly account for the reduced T cell depletion and delayed progression to AIDS.

## Results

### Clinical characteristics of study participants

We included 14 elite controllers, 10 viremic controllers, and 7 treated HIV non-controllers from the elite study [13]. The participant characteristics are summarized in Table 1 while the detailed demographic and clinical characteristics of study participants are in additional Table 1. All HIV controllers were in care for at least five years without ART. None of the HIV controllers had been initiated on ART during blood collection or previously. The comparison group comprised treated HIV non-controllers who had suppressed HIV viral load and maintained CD4 + T cell count greater than 500 cells/ul for at least five years. The mean CD4 + T cell count for all HIV controllers was above 500 cells/ul while the viral load was undetectable in elite controllers and lay between 50 and 2000 copies/ml for viremic controllers.

**Table 1:** Summary of characteristics of study participants

	Elite controllers	Viremic controllers	Non controllers
Number	14	10	7
Mean age, years	40±8	44±9	39±5
CD4 cells/ ul	944±195	774±94	884±197
Mean VL/ ml	UD	670±505	192±81
Mean duration in care(years)	7±2	8±2	6±1

UD: Undetectable. A measure of central tendency and variance: (Mean± SD)

### The percentage of CD4 + T cells is comparable between HIV controllers and non-controllers

**Fig. 1:**
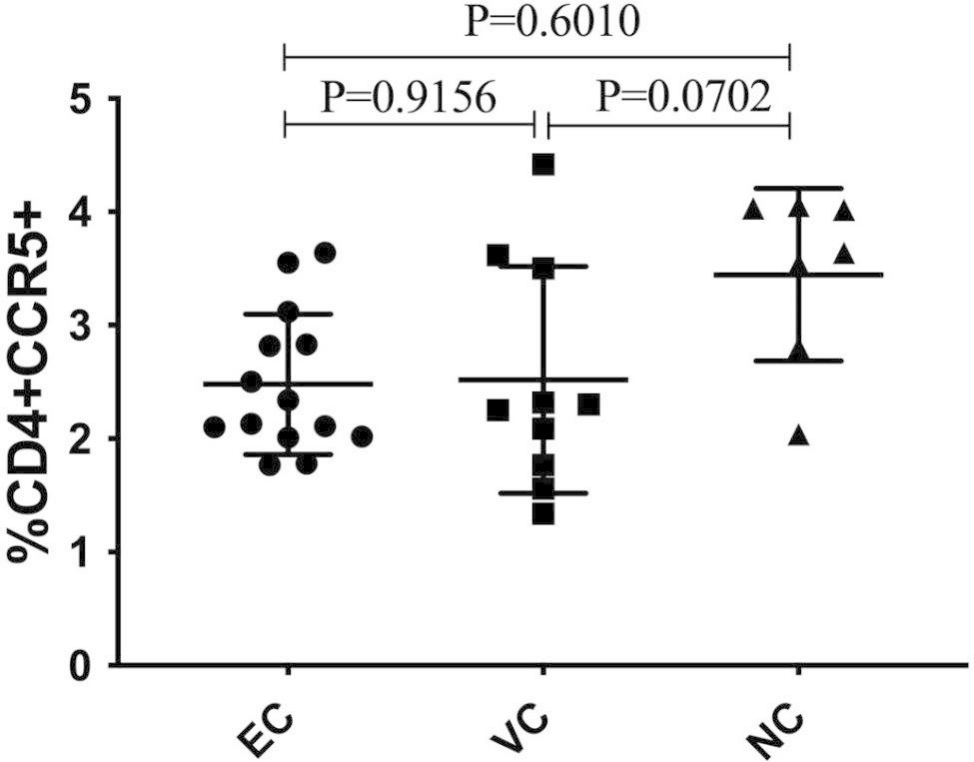
Percentage of CCR5 + CD4 + T cells between HIV controllers and non-controllers. EC: Elite controllers, VC: viremic controllers, and NC: non-controllers. Each point represents one individual

Many studies, including a recent study by Claireaux and colleagues have reported that HIV controllers maintain high CD4 + T cells without ART [6]. To the contrary, non-controllers require ART to inhibit viral replication, CD4 + T cell depletion, and delayed progression to AIDS. In agreement with previous studies, we found no statistical difference between the CCR5 + CD4 + T cell percentages between HIV controllers and HIV-treated non-controllers (Fig. 1; ECs vs. NCs, P = 0.6010; VCs vs. NCs, P = 0.0702).

### HIV controllers have reduced CCR5 expression on CD4 + T cells compared to non-controllers

**Fig. 2:**
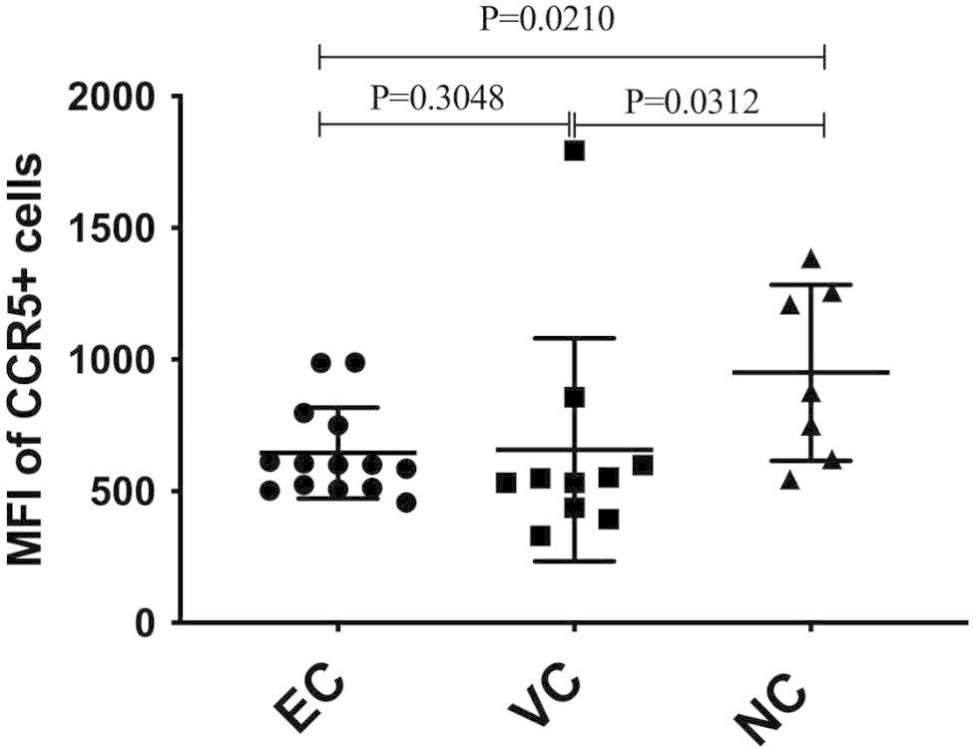
CCR5 densities on CD4 + T cells among HIV controllers compared to non-controllers. EC: Elite controllers, VC: viremic controllers, and NC: non-controllers. Each point represents one individual

How CD4 + T cells from Uganda HIV controllers avoid infection and depletion upon encountering the virus remains incompletely understood. Recently, Claireaux et al. reported that HIV controllers maintain high CD4 + T cell counts because their T cells have reduced CCR5 expression restricting viral entry and cell depletion [6, 24]. We evaluated whether reduced CCR5 expression could account for this phenotype among Ugandan HIV controllers. We found that HIV controllers had significantly reduced CCR5 expression compared to treated HIV non-controllers (Fig. 2; ECs vs. NCs, P = 0.0210; VCs vs. NCs, P = 0.0312).

### Mutations identified in the promoter region of the *CCR5* gene

Many mutations within the *CCR5* gene including delta 32 bp deletion, either abrogate or reduce CCR5 expression among HIV controllers [4–7]. Other studies have reported that increased expression of β-chemokine ligands upon high-avidity antigen/TCR interactions contributes to autocrine CCR5 downregulation in HIV controllers without *CCR5* mutations [6, 24]. Whether these factors account for reduced CCR5 expression among Ugandan HIV controllers remains to be determined. We evaluated whether *CCR5* promoter polymorphisms contribute to reduced CCR5 expression among Ugandan HIV controllers because the delta 32 bp mutation previously reported to impair CCR5 expression is rare in Africa [25, 26]. Many mutations within the promoter region have been reported to reduce CCR5 expression [27–30]. We found rs1799987 SNP (-2459 A/G) predominantly among HIV controllers (71.4% of the elite controllers and 60% of the viremic controllers) while rs41469351 (-2132 C/T) SNP was predominantly found among the treated HIV non-controllers (57.1%). From previous studies, rs1799987 SNP has been reported to reduce CCR5 expression while rs41469351 has been associated with increased perinatal HIV transmission and vaginal HIV shedding [27, 30–33].

## Discussion

The reasons underlying delayed HIV progression among Ugandan HIV controllers remain largely undetermined [13, 14, 34]. The mechanisms for HIV control are heterogenous with no one mechanism universal to all HIV controllers [17, 18, 35–37]. Amanya and colleagues reported that a subset of Ugandan HIV controllers carries rs10838525 SNP (R136Q variant) within the *Trim 5a* encoding gene [21]. This SNP increases the affinity of TRIM 5a for HIV capsid thus enhancing interference with viral decapitation [23, 35] suggesting that genetic factors contribute to HIV control mechanisms. In this study, we show that HIV controllers significantly downregulate their expression of CCR5 on CD4 + T cells compared to non-controllers despite their similar levels of CD4 + T cells. Further, we report that rs179998, a SNP known to reduce CCR5 expression may be partly responsible for this low expression because we found it to be highly predominant in the HIV controllers in our study.

Many studies have previously reported that decreased CCR5 expression reduces HIV viral entry and replication [6, 24, 38, 39]. Gonzalo-Gil and colleagues have previously showed that CD4 + T cells from HIV controllers are resistant to infection with the R5 tropic HIV and that this phenotype is reversed by introducing CCR5 [24]. Furthermore, Claireaux and colleagues have recently reported that Gag-specific CD4 + T cells from HIV controllers have downregulated CCR5, significantly decreasing their susceptibility to R5 tropic HIV entry [6]. In agreement with Claireaux and colleagues, we also found that Ugandan HIV controllers have significantly reduced CCR5 expression compared to non-controllers (Fig. 2) but with comparable percentages of CD4 + T cells (Fig. 1). We believe HIV controllers maintain high CD4 + T cell counts partly because their T cells have significantly reduced CCR5 expression which protects them from infection and depletion.

Impaired CCR5 expression can be due to functional or genetic factors [6]. Claireaux and colleagues have shown that a subset of controllers carry biallelic mutations for example, Q280P, which significantly reduce CCR5 expression [6]. Many other mutations have been reported to impair CCR5 expression [28–30, 40, 41]. Our focus was on mutations within the promoter region that have been previously reported to reduce CCR5 expression and shown to delay HIV-1 disease progression. We found rs1799987 SNP and rs41469351 SNP among HIV controllers and treated HIV non-controllers respectively. In agreement with McDermott et al. and Knudsen et al., rs1799987 SNP was more pronounced among individuals that delayed progression to AIDS [27, 42]. In several other studies, rs1799987 SNP has been associated with significantly reduced in vitro promoter activity, CCR5 expression, and HIV propagation compared to wild-type ORF [27, 30, 43–48].

On the other hand, treated HIV non-controllers predominantly had the rs41469351 SNP. Kostrikis et al. and John et al. have reported that infants with rs41469351 SNP have enhanced susceptibility to HIV − 1 infection [31, 32]. Kostrikis et al. reported that this mutation is associated with increased perinatal HIV transmission. To the contrary, John et al. and Singh et al. did not find such a relationship [32, 33]. However, John et al. reported that women with the rs41469351 SNP had a 3.1-fold increased risk of death during the 2-year follow-up period and significantly increased vaginal shedding of HIV-1–infected cells [32]. We believe rs41469351 SNP influences HIV infection, but its impact on CCR5 expression and viral entry remains to be determined.

Because mutations in the *CCR5* gene partly account for the reduced CCR5 expression among HIV controllers, other mechanisms that could account for this phenotype have been explored. Claireaux and colleagues have recently reported that increased expression of β-chemokine ligands upon high-avidity antigen/TCR interactions contributes to autocrine CCR5 downregulation in controllers without *CCR5* mutations [6]. We believe a similar mechanism could explain the reduced CCR5 expression among the remaining subset of Ugandan HIV controllers who do not carry mutations associated with reduced CCR5 expression.

## Conclusion

HIV controllers maintain comparable CCR5 + CD4 + T cells to treated HIV patients, and this could be partly because of the significantly reduced CCR5 expression. Reasons for the reduced CCR5 expression among Ugandan HIV controllers remain to be determined, but we identified rs1799987 SNP among a subset of controllers. This SNP has been previously reported to reduce CCR5 expression.

### Limitations of the study

The study population was constrained because participants were recruited at a time when the test and treat policy was being initiated in Uganda. All individuals that were initiated on treatment by the time of enrolment were excluded regardless of their previous HIV control status. Furthermore, we were unable to include healthy HIV unexposed or exposed controls, but previous studies show that HIV controllers have reduced CCR5 expression compared to both non-controllers and healthy uninfected individuals [24, 49, 50].

## Methods

### The aim, research design, and setting of the study

This laboratory-based cross-sectional study was conducted to characterize CCR5 expression between HIV controllers and treated HIV non-controllers in Uganda. We utilized PBMCs from the Elite study that recruited participants from Makerere University Joint Aids Program (MJAP) ISS clinic between 2016 and 2018 in Uganda [13]. The immunology experiments were conducted at Makerere University College of Health Sciences, Molecular and Immunology Laboratory. The molecular biology experiments were conducted at the Center for AIDS Research (CFAR) laboratory, Joint Clinical Research Center in Kampala, Uganda.

### Participant characteristics

The study utilized PBMCs collected by the elite study [13]. Before implementing the test and treat policy, the Elite study recruited HIV+, ART-naive individuals aged 18 years and older who were under care for at least five years at Makerere University Joint AIDS Program (MJAP), Mulago ISS clinic in Kampala, Uganda [13]. Patient records were reviewed, and individuals that maintained baseline viral load below 2000 copies per milliliter and serial CD4 counts greater than 500 cells/ml in the absence of ART were recruited. The study participants were classified into the elite (< 50 copies/ml) and viremic controllers (50–2000 copies/ml) based on their HIV viral load. The control group included treated HIV + non-controllers who suppressed HIV for at least five years. The study excluded individuals with hemoglobin of 10 mg/dL or active opportunistic infection. The School of Biomedical Sciences, Higher Degrees Research and Ethics Committee (SBS HDREC), Makerere University (SBS-604) approved the research.

### Laboratory methods

#### Sample processing and thawing

PBMCs were retrieved from liquid nitrogen and immediately thawed in a water bath set at 37 °C. After that, they were transferred into 10 ml of R-10 media and centrifuged at 1500 rpm for 10 min. The supernatant was decanted, and the pellet was resuspended in 5 ml R-10 media (10% FBS, 1% Pen-strep, 1% L-Glutamine, 1% Hepes Buffer) RPMI) for counting. The cells were stained with trypan blue and counted using an automatic cell counter (Invitrogen, Carlsbad, California, USA).

### CCR5 phenotyping

CD4 T cells were isolated from PBMCs retrieved from liquid nitrogen using the EasySep™ Human Isolation Kit (Stem Cell Technologies) according to the manufacturer’s protocol. The isolated cells were assessed for purity using flow cytometry. Briefly, cells were stained with anti-CD3 and anti-CD4 and ran on the BD FACS Canto II (BD Biosciences, Franklin Lakes, New Jersey, USA). Samples with an average purity of ≥ 95% were considered for stimulation. CD4 + T cells were then stimulated with anti-CD3 (eBioscience Clone CD28.2) and anti-CD28 (eBioscience clone OKT3) at a 5 µg/ml concentration each and incubated for 48 h at 37^0^ C in a CO_2_ incubator. After incubation, cells were washed and stained for CCR5 phenotyping monoclonal antibodies. Cells were stained with Zombie aqua and antibodies against CD3, CD4, and CCR5 (BD bioscience, San Jose, CA, USA) and were acquired on an eight-color FACS CANTO II (BD Biosciences, San Jose, CA, USA). Data were analyzed using FlowJo version 10.1 (San Carlos, CA, USA). The gating strategy used is shown in Additional Fig. 1.

### DNA extraction and PCR amplification

DNA was extracted from CD4 + T cells using the QIAamp DNA mini-Kit (Qiagen, Inc., Valencia, CA, USA) per the manufacturer’s instructions. The *CCR5* promoter region was amplified as described by Picton et al. [51]. Briefly, a PCR master mix was prepared with high fidelity Super script III platinum Taq polymerase (Invitrogen, Carlsbad, CA, USA), 2X reaction buffer, 5 Mm MgCL2 and primers (Forward- 5′CCAAGCACCAGCAATTAGC3′ and Reverse 5′TGCCACCACAGATGAATGTC3′) developed using GenBank sequence with accession number U95626. PCR was run with the following cycling conditions; Initial denaturation at 95 °C for 3 min; 31 cycles of denaturation at 95 °C for 30 s, annealing at 60 °C for 30 s, extension at 68 °C for 2.40 min; followed by 68 °C for 7 min. The promoter amplicon size was 2189 base pairs [51].

### Sequencing

PCR amplicons were cleaned using the ExoSAP IT and sequenced using an ABI version 3.1 BigDye Kit (Applied Biosystems, Catalogue no. 4,337,456) and ABI3500xl Genetic Analyzer. Briefly, a master mix was prepared as follows; 0.5 µl Big Dye terminator, 1.75 µl 5X sequencing buffer, 2.5 µl primer (sequences in Additional Table 2), and 4.25 µl water. 9 µl of sequencing master mix was added into each well, where 1 µl of DNA was added. PCR amplification was subjected to thermal cycling as follows: 96 °C for 1 min; 30 cycles of denaturation at 96 °C for 30 s, annealing at 60 °C for 30 s, extension at 68 °C for 2.40 min; followed by 68 °C for 7 min.

### Sanger sequence data analysis

Mutation surveyor version 5.5 (Soft Genetics; Pennsylvania, USA) was used to identify mutations. U95626 and NT_022517 reference sequences were used in assembly [52]. The *CCR5* numbering system was used where the first nucleotide of the translational start site is designated as + 1, and the nucleotide immediately upstream from that is − 1 [53]. A search of the GenBank NCBI SNP database (dbSNP) determined whether polymorphisms detected in this study had been previously reported.

### Statistics

Statistical analysis was performed using GraphPad Prism 7. The Mann-Whitney and Kruskal Wallis test for non-parametric variables facilitated the comparison of differences among groups. P values < 0.05 indicated a significant difference.

## Electronic supplementary material

Below is the link to the electronic supplementary material.

Supplementary Material 1

Supplementary Material 2

Supplementary Material 3

## Data Availability

The datasets used and analyzed during the current study are available from the corresponding author upon reasonable request.

## Abbreviations

CCR5: CC chemokine receptor 5
ART: Antiretroviral therapy
DNA: Deoxyribonucleic acid
HIV: Human immunodeficiency virus
MJAP: Makerere University Joint Aids Program
PBMC: Peripheral blood mononuclear cells
PCR: Polymerase chain reaction
RNA: Ribonucleic Acid
EC: Elite controllers
NC: Non-controllers
LTNP: Long term nonprogressors
