# Supplementary material for: Reduced CCR5 expression among Uganda HIV controllers
Source: Retrovirology. 2023 May 25;20:8. doi: 10.1186/s12977-023-00626-7 (PMC10210444; doi:10.1186/s12977-023-00626-7)
Supplement: Supplementary file 1 — Supplementary Material 1 [file 12977_2023_626_MOESM1_ESM.docx]

**Additional table 1: Demographic and Clinical characteristics of participants**

| **Phenotype** | **Sex** | **CD4 count** | **Duration in Care (Years)** | **Viral Load** |
| --- | --- | --- | --- | --- |
| Elite controller 1 | Female | 1245 | 10 | Undetectable |
| Elite controller 2 | Female | 1008 | 5 | Undetectable |
| Elite controller 3 | Female | 919 | 9 | Undetectable |
| Elite controller 4 | Female | 1188 | 7 | Undetectable |
| Elite controller 5 | Male | 833 | 7 | Undetectable |
| Elite controller 6 | Male | 778 | 9 | Undetectable |
| Elite controller 7 | Female | 1063 | 6 | Undetectable |
| Elite controller 8 | Female | 1036 | 6 | Undetectable |
| Elite controller 9 | Female | 653 | 8 | Undetectable |
| Elite controller 10 | Female | 909 | 5 | Undetectable |
| Elite controller 11 | Female | 1050 | 5 | Undetectable |
| Elite controller 12 | Female | 728 | 6 | Undetectable |
| Elite controller 13 | Female | 1162 | 6 | Undetectable |
| Elite controller 14 | Female | 650 | 9 | Undetectable |
| Viremic controller 1 | Female | 698 | 5 | 280 |
| Viremic controller 2 | Female | 652 | 8 | 1380 |
| Viremic controller 3 | Female | 895 | 10 | 1299 |
| Viremic controller 4 | Female | 805 | 5 | 155 |
| Viremic controller 5 | Male | 732 | 6 | 388 |
| Viremic controller 6 | Female | 852 | 10 | 285 |
| Viremic controller 7 | Male | 897 | 11 | 1220 |
| Viremic controller 8 | Female | 772 | 10 | 243 |
| Viremic controller 9 | Female | 669 | 8 | 782 |
| Viremic controller 10 | Female | 918 | 8 | 1490 |
| Non-controller 1 | Female | 589 | 5 | 10500 |
| Non-controller 2 | Female | 1021 | 8 | 2840 |
| Non-controller 3 | Male | 920 | 6 | 10800 |
| Non-controller 4 | Female | 940 | 5 | 14800 |
| Non-controller 5 | Female | 747 | 5 | 2310 |
| Non-controller 6 | Female | 781 | 8 | 5250 |
| Non-controller 7 | Female | 1192 | 6 | 2840 |
