# Supplementary material for: Reduced CCR5 expression among Uganda HIV controllers
Source: Retrovirology. 2023 May 25;20:8. doi: 10.1186/s12977-023-00626-7 (PMC10210444; doi:10.1186/s12977-023-00626-7)
Supplement: Supplementary file 2 — Supplementary Material 2 [file 12977_2023_626_MOESM2_ESM.docx]

**Supplementary table 1: Primers used in sequencing of the CCR5 promoter 1 region**

| **Primer** | **Binding position** |
| --- | --- |
| Forward F 5′CCAAGCACCAGCAATTAGC3′ | 58105 – 58122 |
| Reverse R 5′TGCCACCACAGATGAATGTC3′ | 60293 – 60274 |
| Forward IFS 5′TTGCTGTTTGGGGTCT3′ | 58471 – 58486 |
| Forward F1 5′GAGTGGAGAAAAAGGGGG3′ | 59013 – 59030 |
| Reverse R1 3′AGAATAGATCTCTGGTCTGAAA5′ | 59375 – 59354 |
