# Supplementary figures and images for: Reduced CCR5 expression among Uganda HIV controllers
Source: Retrovirology. 2023 May 25;20:8. doi: 10.1186/s12977-023-00626-7 (PMC10210444; doi:10.1186/s12977-023-00626-7)

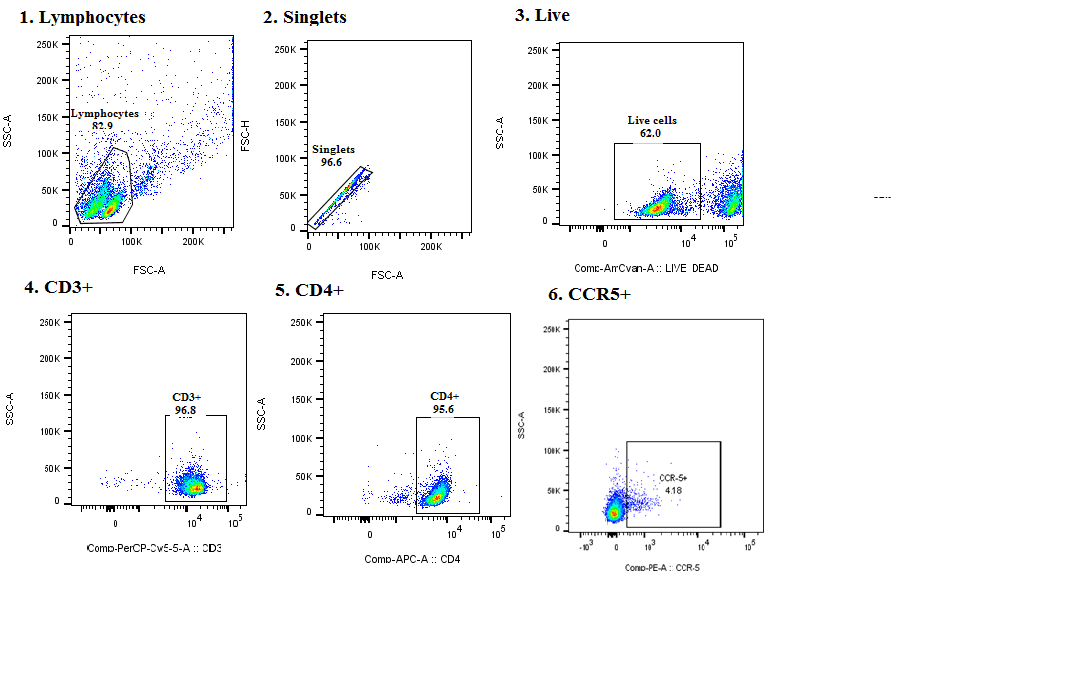

Supplement: Supplementary file 3 — Supplementary Material 3 [file 12977_2023_626_MOESM3_ESM.jpg]
